# Multisystem impairment in South African adolescents with Perinatally acquired HIV on antiretroviral therapy (ART)

**DOI:** 10.1002/jia2.25386

**Published:** 2019-08-23

**Authors:** Lisa J Frigati, Karryn Brown, Sana Mahtab, Leah Githinji, Diane Gray, Liesl Zühlke, Peter Nourse, Dan J Stein, Jaqueline Hoare, Mark F Cotton, Landon Myer, Heather J Zar

**Affiliations:** ^1^ Department of Paediatrics and Child Health University of Cape Town Cape Town South Africa; ^2^ Department of Paediatrics and Child Health Stellenbosch University Cape Town South Africa; ^3^ Division of Epidemiology and Biostatistics School of Public Health and Family Medicine University of Cape Town Cape Town South Africa; ^4^ Department of Psychiatry and Mental Health University of Cape Town Cape Town South Africa; ^5^ SAMRC Unit on Child and Adolescent Health University of Cape Town Cape Town South Africa

**Keywords:** perinatally HIV‐positive adolescents, multisystem morbidity, antiretroviral therapy, chronic disease, Sub‐Saharan Africa

## Abstract

**Introduction:**

Adolescents with perinatally acquired HIV (PHIV) are at risk of chronic disease due to long‐standing immune suppression, HIV disease and antiretroviral therapy (ART) exposure. However, there are few data on multisystem disease in this population. We investigated the overlapping burden of neurocognitive, cardiovascular, respiratory and/or renal impairment among PHIV positive (PHIV+) adolescents.

**Methods:**

In this cross‐sectional analysis, participants aged 9 to 14 years on ART for >6 months were recruited from seven sites across Cape Town from July 2013 through March 2015, together with age‐matched HIV‐negative (HIV‐) adolescents. Impairment at enrolment was assessed across neurocognitive functioning (using the youth‐International HIV Dementia Scale); cardiac function (echocardiogram abnormality); respiratory function (abnormal spirometry) and renal function (abnormal glomerular filtration rate).

**Results and Discussion:**

Overall, 384 PHIV+ and 95 HIV‐ adolescents were included (mean age, 11.9 years; 49% female). Median age of ART initiation was 4.2 years (IQR: 1.7 to 7.6) and median CD4 count was 709 (IQR: 556 to 944) with 302 (79%) of PHIV+ adolescents virologically suppressed. Abacavir and Zidovudine were the most commonly used nucleoside reverse transcriptase inhibitors (NRTIs) with 60% of adolescents on non‐nucleoside reverse transcriptase inhibitors (NNRTI) and 38% on a protease inhibitor (PI). Among PHIV+ adolescents, 167 (43.5%) had single system impairment only, 110 (28.6%) had two systems involved, and 39 (10.2%) had three or four systems involved. PHIV+ participants had more 2‐system and 3‐system impairment than HIV‐, 110 (28.6%) versus 17 (17.9%), *p *=* *0.03 and 39 (10.2%) versus 3 (4.3%), *p *=* *0.03. PHIV+ participants who had failed a year of school (73.8% vs. 46.4%, *p *=* *0.00) and with a viral load >1000 copies/mL at enrolment (16.8% vs. 8.1%, *p *=* *0.03) were more likely to have dual or multisystem impairment. Of those with cardiac impairment, 86.7% had an additional system impaired. Similarly, in those with neurocognitive impairment, almost 60% had additional systems impaired and of those with respiratory impairment, 74% had additional systems impaired.

**Conclusions:**

Despite relatively early ART initiation, there is a substantial burden of multisystem chronic impairment among PHIV+ adolescents. This phenomenon needs to be further explored as this population ages and begins to engage in adult lifestyle factors that may compound these impairments.

## 1 Introduction

There are 2.1 million adolescents aged 10 to 19 years living with HIV, the majority of whom live in sub‐Saharan Africa and have perinatally acquired infection 1, 2. This population is growing as most children on antiretroviral therapy (ART) are surviving into adolescence, due to increased access and earlier initiation of ART 3. Single system morbidity in perinatally HIV‐positive (PHIV+) adolescents is well described but there is limited data on multimorbidity in this population.

Cardiac, respiratory, neurocognitive or chronic renal impairment are common long‐term sequelae of perinatal HIV infection 4, 5, 6, 7. PHIV+ adolescents in the United States (USA) and Europe have high rates of chronic lung disease, kidney disease and neuropsychiatric problems 8. In sub‐Saharan Africa, however, diagnosis and initiation of ART occur much later than in the USA, with the median ages at first visit and at ART initiation of 7.1 and 7.9 years compared to 0.7 and 0.9 years respectively 9. HIV+ children in sub‐Saharan Africa are therefore at greater risk of chronic morbidity due to untreated HIV in childhood, long‐standing immune suppression and associated infection, suboptimal ART formulations and regimens or lack of access to care 10. The interplay between these factors along with chronic inflammation result in PHIV+ adolescents being at risk for multisystem impairment 11.

Single system morbidity has been described in resource limited settings, mostly focusing on cardiac or chronic lung disease 12, 13. A recent study from Asia showed that infectious HIV‐related morbidity was more common in younger adolescence (10 to 14 years of age) with a trend toward non‐infectious and treatment‐ related morbidity in later adolescence, however, this study did not specifically explore chronic or multisystem morbidity 14.

Despite the high prevalence of single system morbidity, there are surprisingly few data on prevalence of and risk factors for multisystem involvement in PHIV+ adolescents on ART, especially in Africa. Multisystem morbidity may be associated with worse clinical outcomes, increased healthcare utilization and more difficulty in adhering to ART due to a high pill burden. Many studies of single organ impairment are limited by small sample sizes, with no comparison group of HIV‐ adolescents and many are from the pre‐ART era. As growing numbers of PHIV+ adolescents present to overburdened health systems in resource limited countries, optimizing strategies to best care for them is crucial. The aim of this study was to investigate the prevalence of and risk factors for overlapping multisystem (neurocognitive, cardiovascular, respiratory and renal) impairment in PHIV+ adolescents in the Cape Town Antiretroviral Cohort (CTAAC).

## 2 Methods

### 2.1 Study population

This was a cross‐sectional study of PHIV+ children and adolescents enrolled in CTAAC, a longitudinal cohort study in Cape Town, South Africa. Children between 9 and 14 years on ART for more than six months were enrolled from seven sites in the Western Cape Province, South Africa with age matched HIV‐ youth of similar ancestry from July 2013 to March 2015. Children and adolescents between the ages of nine to fourteen years were considered to be perinatally infected 15. Ethical approval was given by the Faculty of Health Sciences, University of Cape Town and Stellenbosch University, Human Research Ethics Committee (051/2013). Parents gave informed consent and assent was obtained from all adolescents. All participants knew their HIV status as a pre‐requisite to study enrolment.

**Figure 1:**
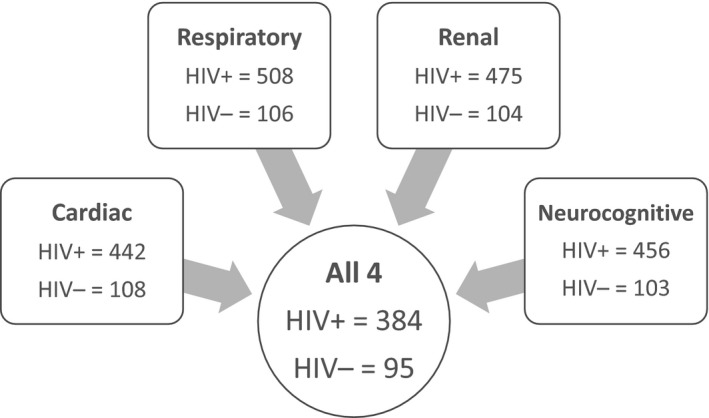
Participants with completed study measures from a total of 625 participants

For this analysis, we included only those participants with complete respiratory, cardiac, neurological and renal assessments from the enrolment visit (Figure 1).

### 2.2 Study measures

#### 2.2.1 Sociodemographic data and other health information

Routine sociodemographic data were collected at enrolment and each participant's clinical record was reviewed at their primary treatment facility.

Participants were screened for cardiac and respiratory symptoms such as wheeze, cough, shortness of breath and a validated respiratory questionnaire derived from the International Study of Asthma and Allergies in Childhood study was performed at enrolment 16.

A physical examination including Tanner staging, WHO HIV staging, blood pressure (BP) and anthropometry was performed at enrolment. Body Mass Index (BMI) was calculated as weight in kilogrammes divided by height in metres squared (kg/m^2^). BMI was classified according to WHO reference standards 17. BP was measured using an electronic sphygmomanometer (Spot Vital Signs, Welch Allyn, New York, NY, USA). All anthropometric measures were performed by one of two trained study nurses to ensure standardization of measures.

Laboratory measures performed at enrolment included viral load (COBAS Ampliprep system; Roche Molecular Systems, Branchburg, NJ, USA) and CD4 count (Beckman Coulter^®^, Brea, CA, USA) in HIV+ participants. Abnormal total cholesterol (TC), high‐density lipoprotein (HDL), and low‐density lipoprotein (LDL) were defined as >5.18, <1.03 and >3.37 mmol/L respectively. Abnormal triglycerides were defined as >2.85 mmol/L if age <10 years or >3.89 mmol/L if age ≥ 10 years at the time of baseline investigations 18. The Homeostatic Model Assessment (HOMA) (fasting insulin [mIU/L]×fasting glucose [mmol/L] divided by 22.5) was used to assess insulin resistance (IR) 19.

#### 2.2.2 Cardiac measures

Echocardiograms were performed by two trained research echocardiographers using either a Philips iE33 or CX50 (Phillips, Eindhoven, The Netherlands) using standardized techniques. All echocardiograms were interpreted by a single paediatric cardiologist and a random subset was read by a second blinded paediatric cardiologist. Left ventricular shortening fraction was measured by M‐mode and ejection fraction was derived using standard methods. The ejection fraction was also measured using the modified Simpson's method 20. Left ventricular diastolic function was measured using Doppler assessment of mitral inflow. Tissue Doppler techniques were used to measure mitral annular velocity. Tricuspid annular plane excursion was measured using M‐mode 20. Pulmonary artery pressures (systolic and diastolic) were estimated using standard continuous and pulse wave Doppler methods.

Cardiac dimensions were assessed either using direct measurement of 2‐D images or M‐mode recordings.

Body surface area (BSA) was estimated using the Mosteller formula 21. Echocardiographic structural parameters were expressed as raw means as well as a deviation from the BSA‐corrected mean (z‐scores), based on normal values 22.

Cardiac impairment was defined as any one or more of the following six findings: (i) Left ventricular (LV) hypertrophy defined as a LV mass >88 g/m^2^ for females and >102 g/m^2^ for males 23 (ii) LV systolic dysfunction defined by LV shortening fraction (LVSF) ≤25% 20, (iii) LV diastolic dysfunction defined by the early to late ventricular filling ratio (E/A ratio) 24, (vi) right ventricular (RV) systolic dysfunction determined using a tricuspid annular plane systolic excursion (TAPSE) z‐score <2 25, (v) fractional RV area change (FAC) ≤34% 26 and (vi) mean pulmonary arterial pressure (mPAP) >25 mmHg 27.

#### 2.2.3 Respiratory measures

Spirometry was done using the NDD EasyOne Pro LAB^®^ (NDD, Switzerland). Testing adhered to the American Thoracic Society/European Respiratory Society (ATS/ERS) guidelines 28, 29, 30. Lower limit of normal (LLN) for spirometry outcome variables was calculated using the African‐American reference cohort in global lung initiative (GLI) software, −1.64 standard deviations (SD) below the mean 31. Lung function testing was deferred if the participant had an acute respiratory illness.

Respiratory impairment was defined as a forced expiratory volume (FEV_1_) below the lower limit of normal (FEV_1_) <LLN) or FEV_1_/forced vital capacity (FEV_1_/FVC<LLN) 32.

#### 2.2.4 Neurocognitive measures

The youth‐International HIV Dementia Scale (y‐IHDS), a sensitive screening test for neurocognitive disorders, was used to screen for cognitive impairment 33. The y‐IHDS is a 3‐part test that includes timed finger tapping, a time alternating hand sequence test and a two‐minute delayed recall of four words 34. Each participant is asked about a history of repeating a grade/grades at school, with one point subtracted from the total score for positive response. The test was conducted in the participant's home language by trained study doctors.

Neurocognitive impairment was defined as a y‐IHDS less than or equal to 10 33.

### 2.3 Renal measures

Enrolment blood was taken to assess creatinine and standing height was measured using a stadiometer with a moveable headboard in centimetres.

Serum creatinine was measured in μmol per litre by the enzymatic method. The modified Schwarz formula was used to estimate glomerular filtration rate (GFR) 35. Renal impairment was defined as glomerular filtration rate (GFR) below 90 mL/min/1.73 m^2^.

### 2.4 Statistical analysis

#### 2.4.1 Primary outcomes

The primary outcomes were the number of participants with single, dual and multisystem impairment.

Single, dual and multisystem impairment were defined as having impairment of only one (single), only two (dual) or three or more (multisystem) of the following systems: cardiac, respiratory, neurological or renal.

Baseline variables and outcomes for PHIV + and HIV− adolescents were compared using t‐tests, Wilcoxon and chi‐square tests as appropriate. Among PHIV+ participants, logistic regression was performed to evaluate factors associated with having dual or multisystem impairment. Covariates considered for associations with multisystem impairment included anthropometry, a history of having had Tuberculosis (TB), HIV laboratory parameters, and duration and type of ART.

Statistical analysis was performed using Stata version 14.1. StataCorpInc. College Station, TX, USA.

## 3 Results

Four hundred and seventy‐nine participants (384 PHIV+ and 95 HIV−) had complete respiratory, cardiac, neurological and renal assessments from the enrolment visit.

### 3.1 Characteristics of participants

Of 479 participants, 384 (80%) were PHIV+ and 95 (20%) were HIV−. The mean age was 11.9 years (SD 1.6); 234 (49%) were female and 456 (95.2%) were Black African. PHIV+ participants were more likely to have a history of TB treatment (*p *≤ 0.01), a history of asthma (*p *=* *0.03), or to have failed a grade at school (*p *≤ 0.01) compared to HIV−. Respiratory rate and blood pressure were significantly different although these differences were not clinically important. PHIV+ participants also had a lower BMI, height and were more stunted than HIV‐ participants (*p *≤* *0.01 for all). Almost half (46.4%) PHIV+ participants were prepubertal (Tanner Stage 1) versus 32.6% of HIV‐ participants, *p *≤* *0.01. Lipid abnormalities were more frequent in PHIV+ participants with 56 (14.6%) versus 3 (3.2%) having hypercholesterolaemia, *p *≤* *0.01 (Table 1).

**Table 1:** Clinical and laboratory characteristics of study participants

	PHIV+ (n = 384)^a^	HIV− (n = 95)	*p*‐value
Clinical characteristics			
Age (years)	12.0 (1.7)	11.8 (1.6)	0.28
Female	183 (47.7)	51 (53.7)	0.29
Black African	361 (94.0)	95 (100)	0.01
Previous TB treatment	234 (61.6)	2 (2.2)	0.00
Hospital admissions in past 12 months	8 (2.1)	1 (1.1)	1.00
History of wheeze	43 (11.2)	6 (6.3)	0.19
History of asthma diagnosis	49 (12.8)	4 (4.4)	0.03
History of dyspnoea	12 (3.1)	2 (2.1)	1.00
History of cough	52 (13.7)	8 (8.6)	0.23
History of failing a grade at school	219 (57.0)	33 (34.7)	0.00
BMI (kg/m^2^)	17.1 (15.9 to 18.8)	18.8 (16.6 to 21.5)	0.00
Height	140.6 (10.4)	144.6 (11.1)	0.00
Stunting	101 (26.3)	4 (4.2)	0.00
Puberty			
Pre‐pubertal (Tanner stage 1)	175 (46.4)	31 (32.6)	0.02
Pubertal (Tanner stage 2 to 5)	202 (53.6)	64 (67.4)	
Laboratory measures			
Triglycerides	0.9 (0.7 to 1.1)	0.7 (0.5 to 0.8)	0.00
Hypertriglyceridaemia	12 (3.1)	1 (1.1)	0.48
Total cholesterol	4.1 (0.8)	3.8 (0.7)	0.00
Hypercholesterolaemia	56 (14.6)	3 (3.2)	0.00
LDL	2.2 (0.7)	2.0 (0.6)	0.02
HDL	1.5 (1.3 to 1.7)	1.40 (1.2 to 1.7)	0.28
HOMA	2.0 (1.3 to 3.0)	1.9 (1.2 to 3.6)	0.97
Log HOMA	0.3 (0.3)	0.3 (0.4)	0.46
Viral load (copies/mL)			
<50	302 (79.0)	‐	‐
50 to 1000	37 (9.7)	‐	‐
>1000	44 (11.3)	‐	‐
CD4 count (cells/mm³)			
<200	8 (2.1)	‐	‐
200 to 499	54 (14.1)	‐	‐
≥500	320 (83.8)	‐	‐
WHO HIV staging			
Stage I	28 (7.7)	‐	‐
Stage II	38 (10.4)	‐	‐
Stage III	217 (59.4)	‐	‐
Stage IV	82 (22.5)	‐	‐
Age at initiation of ART (years)			
Median age	4.2 (1.7 to 7.6)	‐	‐
0 to 2	151 (40.1)	‐	‐
3 to 5	95 (25.3)	‐	‐
6 to 14	136 (34.6)	‐	‐
Current ART regimen			
2 × NRTI + NNRTI	225 (60.0)	‐	‐
2 × NRTI + PI	144 (38.0)	‐	‐
Other	8 (2.1)	‐	‐

All continuous variables expressed as median (interquartile range) or mean (SD) and categorical variables as number (%). ART, antiretroviral treatment; BMI, body mass index; HDL, high‐density lipoprotein cholesterol; HOMA, Homeostatic Model Assessment; LDL, low‐density lipoprotein cholesterol; NNRTI, non‐nucleoside reverse transcriptase inhibitor; NRTI, nucleoside reverse transcriptase inhibitor; PI, protease inhibitor; WHO, World Health Organization.

a Total number may vary according to missing data for each variable.

PHIV+ participants initiated ART at a median age of 4.2 years (IQR: 1.7 to 7.6) with a median duration of ART of 7.9 years (IQR: 4.7 to 9.5). The median CD4 count was 709 (IQR: 556 to 944) with 79% of HIV+ participants having a viral load of less than 50 copies/mL.

Two hundred and twenty‐five (60%) participants were on a non‐nucleoside reverse transcriptase inhibitor (NNRTI) based regimen and 144 (38%) were on a protease inhibitor (PI)‐based regimen with only eight (2.1%) on an alternate regimen (either lamivudine monotherapy or a combination of darunavir/ritonavir and raltegravir).

PHIV+ participants had more cardiac (46.1% vs. 33.7%, *p *=* *0.03), respiratory (27.1%) versus 14.7%, *p *=* *0.01) or neurocognitive impairment (56.3% vs. 45.3%, *p *=* *0.05) than HIV‐ participants. There were few participants with renal impairment (2.3% vs. 2.1%, *p *=* *0.89) in both groups (Table 2).

**Table 2:** Types of impairment (N = 479)

	PHIV+ (N = 384)	HIV− (N = 95)	*p*‐value
Any Impairment			
Any cardiac, renal, respiratory or neurocognitive impairment	316 (82.3%)	68 (72.6)	0.02
Cardiac	177 (46.1)	32 (33.7)	0.03
Renal	9 (2.3)	2 (2.1)	0.89
Respiratory	104 (27.1)	14 (14.7)	0.01
Neurocognitive	216 (56.3)	43 (45.3)	0.05
Single system impairment	167 (43.5)	48 (50.5)	
Cardiac	51 (13.3)	14 (14.7)	0.71
Renal	1 (0.3)	1 (1.1)	0.36
Respiratory	27 (7.0)	8 (8.4)	0.66
Neurocognitive	88 (22.9)	25 (26.3)	0.50
Dual system impairment	110 (28.6)	17 (17.9)	0.03
Renal cardiac	0 (0)	0 (0)	‐
Renal respiratory	0 (0)	0 (0)	‐
Renal neurocognitive	0 (0)	0 (0)	‐
Cardiac respiratory	20 (5.2)	2 (2.1)	0.20
Cardiac neurocognitive	67 (17.5)	13 (13.7)	0.44
Respiratory neurocognitive	23 (6.0)	2 (2.1)	0.20
3 system impairment	39 (10.2)	3 (4.3)	0.03
Renal cardiac respiratory	1 (0.3)	0 (0)	‐
Renal neurocognitive respiratory	0 (0)	0 (0)	‐
Renal neurocognitive cardiac	5 (1.3)	1 (1.1)	1.00
Cardiac neurocognitive respiratory	31 (8.1)	2 (2.1)	0.04
4 system impairment			
Renal respiratory neurocognitive cardiac	2 (0.5)	0 (0)	1.000

**Figure 2:**
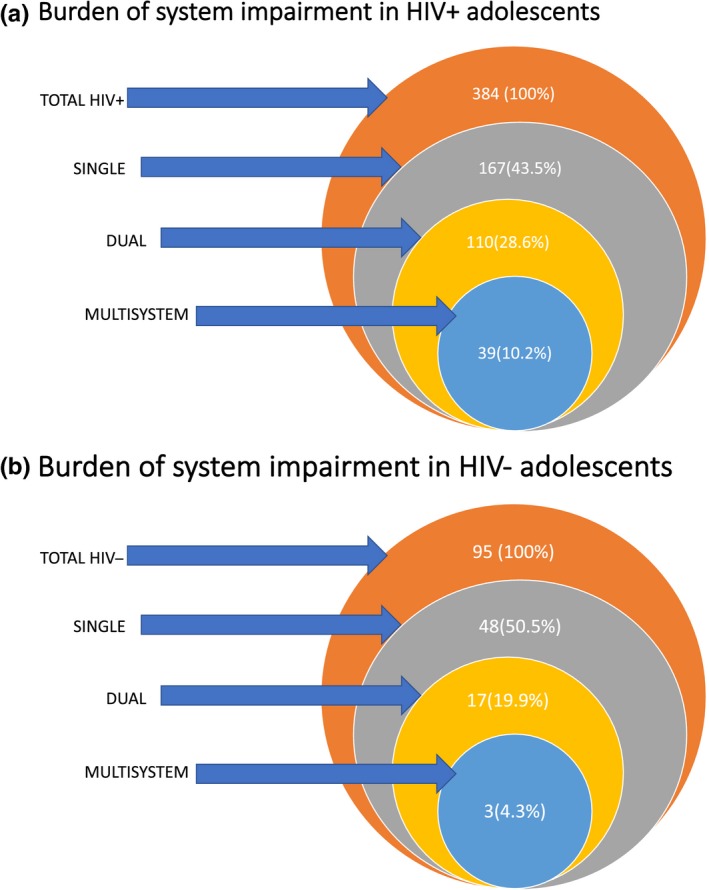
Burden of system impairment in (**A**) HIV+ and (**B**) HIV− adolescents

Two hundred and fifteen (44.9%), of all adolescents had single system impairment only. One hundred and twenty‐seven (26.5%) and 42 (8.8%) had dual and multisystem (three or four systems) involved respectively. PHIV+ participants had more “dual system” (28.6% vs. 17.9%, *p *=* *0.03) and “multisystem” impairment (10.2% vs. 4, *p *=* *0.03) than HIV‐ (Table 2 and Figure 2).

### 3.2 PHIV+ participants

Neurocognitive impairment was the most common type of single system impairment.

Overall, a low TAPSE and FAC, indicative of right ventricular dysfunction, accounted for the majority of echocardiogram abnormalities with 104 (27.1%) and 42 (10.9%) of those PHIV+ adolescents that had cardiac impairment having an abnormal TAPSE and FAC respectively. Left heart dysfunction was rare with only one PHIV+ adolescent having a decreased LVSF and 32 (8.3%) having evidence of left ventricular diastolic dysfunction. No participant had dilated cardiomyopathy and 2 (0.5%) PHIV+ adolescents had raised pulmonary artery pressure. Ninety ‐seven (25.3%) PHIV+ participants had an abnormal FEV1 and 35 (9.1%) had a FEV 1/FVC<LLN.

The most common patterns of dual and multisystem impairment were “cardiac and neurocognitive” and “cardiac, neurocognitive and respiratory” impairment. Only 2 (0.5%) PHIV+ participants had impairment of all four systems. In participants with any impairment, the majority had additional system impairment: Of those found to have cardiac impairment 126/177 (86.7%) had an additional system impaired. Similarly, in those with neurocognitive impairment almost 60% had additional systems impaired and of those with respiratory impairment 74% had additional systems impaired.

In univariate analysis having failed a grade or having a viral load >1000 copies/mL at enrolment, were more common in those with dual or multisystem impairment compared to those with no impairment or single system impairment, 73.8% versus 46.4%, *p *=* *0.00 and 16.8% versus 8.1%, *p *=* *0.03 (Table 3). Participants with dual and multisystem disease were also older 12.2 years versus 11.8 years, *p *=* *0.02.

**Table 3:** No impairment or single system impairment versus dual or multisystem impairment in HIV‐infected participants

	None/single system (n = 235)	Dual/multisystem (n = 149)	*p*‐value
Demographics			
Age (years)	11.8 (1.6)	12.2 (1.6)	0.02
Female	121 (51.5)	62 (41.6)	0.06
History and symptoms			
Wheeze	21 (8.9)	22 (14.8)	0.08
Shortness of breath	7 (3.0)	5 (3.4)	1.00
Cough	26 (11.1)	26 (17.8)	0.07
Repeated a year at school	109 (46.4)	110 (73.8)	0.00
Previous TB	135 (58.2)	99 (66.9)	0.09
Age at ART initiation			
Median age	4.0 (1.7 to 7.7)	4.4 (2.1 to 7.0)	0.60
0 to 2	97 (42.4)	54 (36.7)	0.16
3 to 5	50 (21.8)	45 (30.6)	
6 to 14	82 (35.8)	48 (32.7)	
Current ART regimen			
2 × NRTI + NNRTI	134 (58.3)	91 (61.9)	0.09
2 × NRTI + PI	94 (40.9)	50 (34.0)	
Other	2 (0.9)	6 (4.1)	
Growth measures			
BMI (kg/m^2^)	17.2 (16.0 to 19.0)	16.9 (15.7 to 18.6)	0.12
Height	140.3 (10.0)	141.2 (10.9)	0.41
Stunted	54 (23.0)	47 (31.5)	0.06
Puberty			
Pre‐pubertal (Tanner stage 1)	109 (47.4)	66 (44.9)	0.64
Pubertal (Tanner stage 2 to 5)	121 (52.6)	81 (55.1)	
WHO stage			
Stage I	18 (8.0)	10 (7.2)	0.90
Stage II	25 (11.1)	13 (9.4)	
Stage III	131 (58.0)	86 (61.9)	
Stage IV	52 (23.6)	30 (21.6)	
Laboratory measures			
Viral Load (copies/mL)			
<50	192 (82.1)	110 (73.8)	0.03
50 to 1000	23 (9.8)	14 (9.4)	
>1000	19 (8.1)	25 (16.8)	
CD4 count <200 (cells/mm³)	5 (2.2)	3 (2.0)	0.50

In multivariable analysis failing a grade was associated with dual or multisystem disease (OR = 3.2, CI 2.1 to 5.1, *p *≤* *0.01).

## 4 Discussion

This is the first study to report on multisystem impairment in African PHIV+ children and young adolescents on ART. The study found the prevalence of any cardiac, respiratory or neurocognitive impairment in PHIV+ participants was significantly higher than in HIV− participants. Similar findings of individual system involvement have been reported in other studies of adolescents with perinatally acquired HIV with a high prevalence of cardiac, respiratory, neurocognitive or less commonly renal impairment 5, 36, 37.

Neurocognitive impairment occurring most commonly, is perhaps the most concerning morbidity, impacting on PHIV+ adolescents as it influences all spheres of health including treatment adherence and school performance. The measure used, the y‐IHDS, is only a screening test and cannot definitively diagnose the type or extent of neurocognitive impairment. The score has been validated previously in a subset of this cohort and shown to be sensitive for neurocognitive disorder screening 33. In addition, it is quick and easily performed in busy clinic settings. We found a prevalence of neurocognitive impairment of 56% using y‐IHDS, similar to the 45% of HIV+ youth that met criteria for neurocognitive disorder diagnosis through an extensive battery of neurocognitive tests in the same setting 38. However, in other African settings, the adult IHDS score overestimated the burden of neurocognitive impairment 39. Follow‐up data from our adolescent cohort will be valuable to assess whether the y‐IHDS screening tool correlates with confirmed impairment.

Cardiac impairment was the second most commonly affected system. This was based on echocardiogram parameters reflecting subtle right ventricular dysfunction. The majority of these participants were asymptomatic with no difference between PHIV+ and HIV− participants. These results are consistent with a Spanish study showing subtle cardiac abnormalities found on echocardiogram and no difference in the control population 4. but the authors did not report on right heart dysfunction. Lower TAPSE has been reported in HIV+ young adults but not in HIV− controls 40. Follow‐up of all participants in our study may indicate if these findings are clinically relevant as both TAPSE and FAC are more useful in longitudinal studies 41, 42.

Respiratory single system impairment was reflected by subtle reduction in lung function that may impact on adult lung health. Our findings are consistent with the prevalence of abnormal spirometry, reported as between 24% and 38% in various African adolescent cohorts from Zimbabwe, Malawi or South Africa 32, 43, 44.

Renal impairment was surprisingly rare given the genetic predisposition to HIV nephropathy in Black Africans but may reflect survivor bias as those with more severe kidney disease may have died or already being followed at specialized renal clinics. The prevalence of proteinuria and microalbuminuria, risk factors for chronic kidney disease has been shown to be high in HIV+ adolescents, however, we previously reported no difference in these measurements between PHIV+ and HIV− adolescents in this cohort 45.

Dual system impairment affected about a quarter of participants but multisystem involvement was relatively uncommon. This may reflect that the cohort were ambulatory and relatively well, were on ART for several years and were adherent to therapy. Despite this, there remains a small but clinically significant proportion of adolescents that will need complex clinical services to ensure optimum care. In addition, a significant proportion of those with impairment in one system had another system involved.

Multivariable analysis found that failing a grade at school and age at enrolment was significantly associated with dual or multisystem disease. School failure may be due to neurocognitive impairment or chronic or prolonged illness and hospitalization resulting in missing significant periods of the school year 46. As this was a cross‐sectional study we were unable to infer that multisystem disease had a causal relationship with school failure. Checking for school failure may be helpful in deciding whom to screen for multisystem impairment.

There was no evidence for the association of duration or for the relatively later start of ART and dual or multisystem impairment. A possible explanation for later start of ART not being associated with multisystem impairment is that historically children that were started on early ART prior to the guidelines recommending early start for all were severely ill and this illness may have resulted in multisystem impairment despite early access to ART. In addition, treatment histories were often not available or difficult to interpret. Reasons for switching ART regimens were poorly documented and it was not possible to accurately assess viral suppression prior to study enrolment.

The study is limited by the cross‐sectional analysis that limited the ability to detect longitudinal changes. Only four systems were included, and a more comprehensive assessment of system involvement including hearing, dermatological complications of HIV and musculoskeletal abnormalities may be useful.

An additional limitation is that the measures of impairment that we chose for each system differ in their ability to assess severity of impairment with the y‐IHDS score being a relatively crude estimate of neurocognitive impairment compared to detailed assessment of lung function that is obtained with spirometry. Severity of impairment across different systems thus cannot be directly compared.

## 5 Conclusions

In those PHIV+ adolescents that had one system impaired a significant proportion had another system involved. Although multisystem impairment is relatively rare, a small minority of youth will require clinical attention for complex multisystem issues. Young adolescents with system impairment will need close observation as they transition to adulthood and are increasingly at risk for engaging in adult lifestyle factors such as smoking or recreational drug use. Adult onset diabetes and hypertension may also compound these impairments. Longitudinal follow‐up is needed to ascertain whether system impairment may impact long‐term morbidity.

## Competing interest

All authors declare no competing interest.

## Authors’ contributions

LJF contributed to the initial concept of the paper, did the statistical analysis and wrote the manuscript. KB did statistical analysis. SM contributed towards data management and did statistical analysis. LG, DG performed and interpreted spirometry and were involved in the initial pulmonology concept of CTAAC, LZ read echocardiograms and contributed to the initial cardiology concept of CTAAC, PN gave input on renal measures, DJS and JH gave input on neurocognitive concepts, MFC, LM, HJZ were involved in the initial concept of the paper and obtained funding for CTAAC. All authors have read and approved the final manuscript.
